# Psychometric Properties of the Grit-S in Chinese Nurses

**DOI:** 10.3389/fpsyg.2021.766055

**Published:** 2021-11-02

**Authors:** Changjiu He, Dongmei Wu, Lu Yang, Lei Yang, Yuchuan Yue

**Affiliations:** ^1^The Clinical Hospital of Chengdu Brain Science Institute, MOE Key Laboratory for Neuroinformation, University of Electronic Science and Technology of China, Chengdu, China; ^2^Department of Nursing, The Sixth People’s Hospital of Chengdu, Chengdu, China; ^3^School of Nursing, Chengdu Medical College, Chengdu, China

**Keywords:** grit, scale validation, psychometric properties, nurses, China

## Abstract

Grit, as a positive psychological trait, could affect the stability of nursing workforce and nurses’ physical and mental health continuously. The Short Grit Scale (Grit-S) with fewer items than the original Grit Scale was widely used to measure individual trait-level grit. However, the psychological properties of Grit-S among Chinese nurses have not been verified. A self-designed sociodemographic questionnaire was used to investigate 709 Chinese nurses in the study, and Grit-S, Big Five Inventory-44, Brief Self-Control Scale, 10-item Connor-Davidson resilience scale, and Task Performance Scale were adopted to collect information of grit, personality, self-control, resilience, and work performance. The confirmatory factor analysis, Pearson correlation analysis, hierarchical regression analysis, and multi-group confirmatory factor analysis were conducted to verify the psychometric properties of the Grit-S. The results demonstrated that the Grit-S had sound validity and reliability among Chinese nurse samples and had good measurement invariance across nurses in general hospitals and psychiatric hospitals. The results of this study provide confidence in using the grit measurement among Chinese nurse in the future.

## Introduction

The attention to positive psychology has led to increased interest in its important concepts, and grit is one of them. Individuals with high grit level can maintain their interest and efforts and move forward, regardless of setbacks or difficulties. When exploring why some individuals with similar intelligence quotient are more successful, Duckworth et al. (2007) proposed that trait-level grit may be the key to success and constructed two aspects of grit: maintaining efforts and interest in goals for a long time (Duckworth et al., 2007).

Subsequently, the relationship between grit and personal performance or success has been widely studied, covering a wide range of fields, such as education (Christopoulou et al., 2018; Wei et al., 2019), military (Maddi et al., 2012, 2017), management (Schimschal and Lomas, 2019), economy (Dugan et al., 2019), sports (Meyer et al., 2017; Cormier et al., 2019), and so on. These strands of research have verified that grit could positively predict personal achievements. Moreover, the studies on the relationship between grit and other psychological indicators and healthcare indicators have sprung up over the past few years. Previous studies have found that grit could negatively predict stress, anxiety and depression (Zhang et al., 2018a; Datu et al., 2019; Coleman, 2020), reduce suicidal ideation (Kleiman et al., 2013), and be an important protective factor for adolescent Internet addiction (Borzikowsky and Bernhardt, 2018; Kim et al., 2021). By contrast, grit could positively promote health management skills and health-related quality of life (Traino et al., 2019), improve individual wellbeing (Arya and Lal, 2018; Schimschal et al., 2021) and life satisfaction (Li et al., 2018a).

Considering the two elements of grit: Consistency of interest and perseverance of effort, it is easy to connect it with career and career achievements which are full of challenges, setbacks, difficulties, and require long-term persistence. There is no doubt that healthcare profession, especially nursing, is one of them. Nurses account for more than half of the staff in hospitals (Gutsan et al., 2018) and they are also numerous in the field of primary healthcare (Wolff-Baker and Ordona, 2019). Women still account for the vast majority of nurses in the world (Gutsan et al., 2018). However, nurses have been facing numerous and complex difficulties, especially in hospitals and psychiatric wards, where nurses often confront the uncertain physiological and psychological needs of various patients, complex interpersonal communication problems, insufficient understanding and support of managers, and heavy workload, as well as individual life responsibilities, family problems, and financial needs, especially in the international context of the current global shortage of nurses’ human resources (Zhang et al., 2018b). Therefore, how to reduce nurses’ job burnout and enable nurses to maintain their nursing jobs has become an important proposition around the world.

Studies have shown that grit is a powerful personality trait, which could predict the burnout of healthcare students (Jumat et al., 2020). In the study of nursing staff, it was found that the grit of long-term goals was conducive to long-term work in nursing posts and was negatively correlated with burnout (Seguin, 2019). Moreover, when training the next generation of nursing successors, it is important to consciously cultivate the trait-level grit of nursing students. Previous studies have shown that grit not only helped to improve the adaptability to clinical practice (Baek and Cho, 2020), but also positively predicted the clinical and academic performance of nursing students after controlling for their study years and other demographic factors (Terry and Peck, 2020). In addition, in the field of disaster nursing, the grit of nurses has also been found significant to maintain emergency preparedness, which was also the deep reason for nurses to complete their tasks well in disaster (Tyer-Viola, 2019).

However, the understanding of grit may be different in different cultural backgrounds (Datu et al., 2017; Datu and Mclnerney, 2017). Although the psychometric properties of Short Grit Scale (Grit-S) have been validated among Chinese adolescents (Li et al., 2018b), whether the two-factor structured grit is suitable for Chinese nurses remains to be verified. In addition, compared with nurses in general hospitals, psychiatric nurses have more opportunities to be exposed to psychological-related knowledge and skills training, which may affect their understanding of grit. Therefore, the measurement invariance (MI) across nurses in general hospitals and psychiatric hospitals needs to be studied.

## Materials and Methods

### Participants

Seven hundred and fifty-six nurses who met the criteria were recruited through online advertising and responses from 709 of whom were valid. The response rate was 93.7%. The inclusive criteria were as: (a) registered nurses in the people’s Republic of China; (b) at least 1year working experience in clinical nursing or clinical nursing management; (c) no previous or current diagnosis of mental illness or drug or alcohol dependence; and (d) informed and agreed to participate in the study voluntarily. The exclusion criteria included as: (a) retired nursing staff; (b) nurses on leave during the investigation. The participants gave their informed consent and voluntarily participated in the study. The participants’ characteristics are shown in Table 1.

**Table 1:** Sociodemographic characteristics description.

	Nurses (*N*=709)
Age, mean years and SD (P25, P50, P75)	31.74±7.38 (27, 30, 35)
Length of nursing work, mean years, and SD (P25, P50, P75)	10.67±8.12 (5, 8, 14)
Gender, % (*N*) of female	90.7% (643)
Marital status	
Unmarried	27.9% (198)
Married	68.1% (483)
Divorced	3.7% (26)
Widowed	0.3% (2)
Nursing degree	
Technical secondary school degree	5.2% (37)
College degree	38.1% (270)
Bachelor degree	56.1% (398)
Graduate degree	0.6% (4)
Nurse title	
None	23.0% (163)
Primary	51.6% (366)
Intermediate	24.0% (170)
Vice senior	1.4% (10)
Employed hospital	
General hospital	53.3% (378)
Psychiatric hospital	46.7% (331)

SD, standard deviation; P25, 25 percentile; P50, 50 percentile; and P75, 75 percentile.

### Measures

All tests were conducted in Mandarin Chinese.

#### Sociodemographic Characteristics Data

A self-designed questionnaire was used to collect sociodemographic information, including age, gender, marital status, length of nursing work, nursing degree, nursing title, and employed hospitals’ type.

#### Short Grit Scale

The self-report scale, consisting of two subscales (Consistency of Interest and Perseverance of Effort), was developed and validated by Duckworth and Quinn (2009). A total of eight items were included in the scale, using the 5-point Likert scale (from 1 “not like me at all” to 5 “very much like me”). The higher the factor score, the stronger the corresponding factor. The Chinese version of the Grit-S has good reliability and validity (Li et al., 2018a). The Cronbach’s *α* of the total scale ranged from 0.69 to 0.72 and that of subscales was from 0.58 to 0.71 in the Chinese population (Wang, 2016). The Grit-S was used to assess the grit of all participants in this study and the Cronbach’s *α* of the total scale and subscales was from 0.68 to 0.75.

#### Big Five Inventory-44

The BFI is widely used in personality measurement all over the world (John et al., 1991, 2008; Li et al., 2015). It includes five personality domains: extraversion, agreeableness, conscientiousness, neuroticism, and openness, with a total of 44 items (16 reversed items). It is a 5-point Likert scale, and the item answers range from “strongly disagree” to “strongly agree.” The average score is used to evaluate a certain domain. The higher the average score is, the more obvious the personality of the domain is. The BFI has been proved to have acceptable validity in Chinese population [root mean square error approximation (RMSEA)=0.072–0.099; Zhou, 2010], and the internal consistencies for all personality traits were acceptable (from 0.75 to 0.91; Guo et al., 2021). It is widely used to measure the personality characteristics of nurses in China (Xia et al., 2013; Yu et al., 2019). In this study, BFI-44 was used to measure participants’ personality and to test the convergent validity and criterion-related validity of the Grit-S.

#### Brief Self-Control Scale

The scale, which is used to assess individual self-control, was developed by Tangney et al. in 2004. The brief version (13 items) had high correlation (*r*=0.92–0.93) with the total version (36 items). The items were rated on a 5-point scale, with responses ranging from “not at all like me” to “very much like me”(Tangney et al., 2004). The higher the score of Brief Self-Control Scale (BSCS), the stronger the individual self-control. The Cronbach’s *α* of BSCS was 0.862, and Cronbach’s *α* of each dimension ranged from 0.606 to 0.761 in Chinese population (Tan and Guo, 2008). In this study, BSCS was used to assess individual self-control and to test the convergent validity and criterion-related validity of the Grit-S.

#### 10-Item Connor-Davidson Resilience Scale

The 10-Item Connor-Davidson Resilience Scale (CD-RISC-10) is a unidimensional dimension scale to measure resilience, which was revised from the CD-RISC (Connor and Davidson, 2003; Campbell-Sills and Stein, 2007). It comprises of 10 items, each rated on a 5-point scale (0–4). The higher the score, the better the individual’s resilience.

The Cronbach’s *α* was 0.85 and the factor loading was from 0.44 to 0.74 which indicated good reliability and construct validity in the original study (Campbell-Sills and Stein, 2007). The CD-RISC-10 also showed excellent psychometric properties with favorable internal consistency, structure, and criterion-related validity in Chinese samples (Wang et al., 2010; Cheng et al., 2020). And 48.641% of the total variance was explained in the sample of Chinese nurse students, the Cronbach’s *α* was 0.851 (Ye et al., 2016). In this study, CD-RISC-10 was used to assess individual resilience and to test the convergent validity and criterion-related validity of the Grit-S.

#### Task Performance Scale

Task Performance Scale (TPS), which measures individual task performance, was from Williams and Anderson (1991) (Bachrach et al., 2007). The seven-point scale has five items (e.g., “*adequately completes assigned duties.*”), and each response ranges from highly disagree to highly agree. Task performance is represented by the average score. The higher the score, the better the task performance. The Cronbach’s *α* of the scale was 0.79 in 304 Chinese and American graduate students (Bachrach et al., 2007). In this study, TPS was used to evaluate participants’ task performance and to test the criterion-related validity of the Grit-S.

### Procedure

The study was ethically approved by the Ethics Committee of Chengdu fourth Hospital and the registration number of the Chinese Clinical Trial Registry is ChiCTR1900020715. Participants were recruited through online advertising. Before investigation, written informed consents were obtained. All questionnaires were self-rated, and participants filled separately. According to the unified guidelines, the investigators clarified the unclear and ambiguous items proposed by the participants during the field investigation. And 111 nurses completed the retest after 3months.

### Data Analyses

The following data analyses were used to verify the psychometric characteristics of Grit-S in Chinese nurses including structural validity, reliability, convergent validity, criterion-related validity, and measurement invariance across nurses working in general hospital and psychiatric hospital.

Confirmatory factor analysis (CFA) and Chi-square test were used to verify the structural validity of Grit-S. CFA which is widely used to test the hypothetical relationship between ordinal variables (such as Likert-type items; Flora and Curran, 2004) was performed with IBM SPSS AMOS 23.0. In this study, CFA was used to verify the structural validity with maximum likelihood estimation method. Moreover, Chi-square test was used to compare the two models (unidimensional model and two-dimensional model).

The reliability which was analyzed by SPSS 25.0 was shown by internal consistency using Cronbach’s *α* (Schweizer, 2011) and the test-retest reliability after 3months. The convergent validity was assessed by two-tailed Pearson correlations between both factors of grit with self-control, resilience, and personality. In order to verify the criterion validity, hierarchical regression analysis was used to explore the influence of age, gender, length of nursing work, personality, and grit on task performance.

In addition, compared with nurses in general hospitals, psychiatric nurses may have different education, training, and work experience, which may affect their understanding of grit. Therefore, it is necessary to test the measurement invariance across general hospital nurses and psychiatric hospital nurses by the multi-group confirmatory factor analysis (MGCFA) which was conducted by lavaan package of R 4.0.5.

## Results

### Confirmatory Factor Analysis

**Figure 1:**
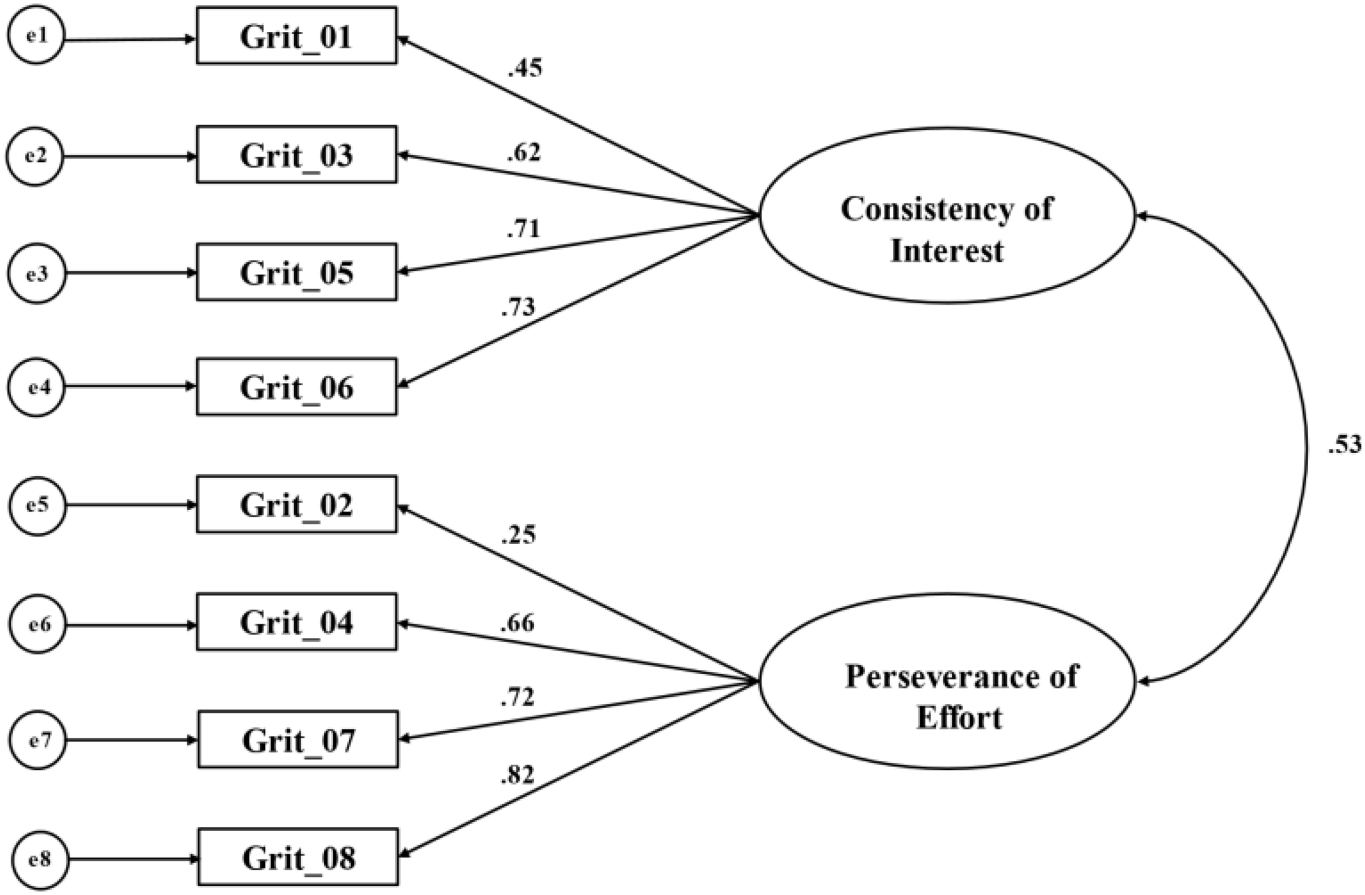
Confirmatory factor analysis (CFA) for the two-dimensional model of Short Grit Scale (Grit-S) with standardized factor loadings.

According to the previous study, the following indices and their criteria were adopted to evaluate the model fitting (Hu and Bentler, 1999), including comparative fit index (CFI) with value greater than 0.95, Tucker-Lewis Index (TLI) with value greater than 0.95, RMSEA with value less than 0.06, and standardized root mean square residual (SRMR) with value less than 0.08. To select the optimal model, two-dimensional model and unidimensional model were compared by chi-square test (see Table 2). And the results supported the former (Figure 1).

**Table 2:** Model fit indices and comparison of Grit-S.

Model	*χ* ^2^	df	TLI	CFI	RMSEA	SRMR	Δ*χ*^2^_(Δdf)_
Two-dimensional	61.132	19	0.953	0.968	0.056	0.0340	
Unidimensional	360.024	20	0.643	0.745	0.155	0.0919	298.892^***^_(1)_

df, degree of freedom; TLI, Tucker-Lewis Index; CFI, comparative fit index; RMSEA, root mean square error approximation; and SRMR, standardized root mean square residual.

*** *p*<0.001 (two tailed).

### Internal Consistency and Test-Retest Reliability

The internal consistency of the scale was evaluated by Cronbach’s *α*. By calculating Cronbach’s *α* of all 709 nurse samples, the Cronbach’s *α* for score of consistency of interest was 0.723, for score of perseverance of effort was 0.682, and for total Grit-S was 0.751. According to the CFA results of this study, the factor load of item 2 was not satisfactory. Cronbach’s *α* of EFF factor after trying to delete item 2 increased to 0.772 and that of Grit-S also increased to 0.772.

To examine test-retest stability, 111 nurse samples (90 female; *M*_age_=31.60, SD=6.603) were retested with Grit-S after 3months. The score correlation coefficient of consistency of interest before and after was 0.805 and that of perseverance of effort and total Grit-S was 0.702 and 0.885, respectively. All test-retest reliability coefficients were acceptable.

### Convergent Validity and Criterion-Related Validity

Table 3 shows the bivariate correlation between the Grit-S factor scores and the scores of self-control, resilience, and personality. The total score of Grit-S and its two factors had a significant positive correlation with self-control and resilience, respectively, and was highly positively correlated with conscientiousness. But in the factor neuroticism of personality, they had a strong negative correlation. These results suggested that the Grit-S was highly correlated with concept-related structure and had acceptable convergent validity.

**Table 3:** Correlation between consistency of interest, perseverance of effort, total Grit-S, self-control, resilience, and personality.

		*M*	SD	1	2	3	4	5	6	7	8	9
1	Consistency of interest	12.47	2.627	—								
2	Perseverance of effort	14.57	2.361	0.377^**^	—							
3	Total Grit-S	27.03	4.141	0.849^**^	0.809^**^	—						
4	Self-control	42.82	6.556	0.588^**^	0.595^**^	0.713^**^	—					
5	Resilience	37.26	5.553	0.368^**^	0.560^**^	0.553^**^	0.484^**^	—				
6	Extraversion	25.16	4.191	0.216^**^	0.309^**^	0.313^**^	0.248^**^	0.477^**^	—			
7	Agreeableness	35.34	3.941	0.322^**^	0.402^**^	0.433^**^	0.478^**^	0.430^**^	0.206^**^	—		
8	Conscientiousness	32.92	4.604	0.410^**^	0.532^**^	0.563^**^	0.648^**^	0.475^**^	0.305^**^	0.495^**^	—	
9	Neuroticism	22.94	4.832	−0.414^**^	−0.334^**^	−0.453^**^	−0.463^**^	−0.561^**^	−0.448^**^	−0.457^**^	−0.492^**^	—
10	Openness	32.10	4.867	0.298^**^	0.349^**^	0.389^**^	0.0319^**^	0.428^**^	0.416^**^	0.283^**^	0.354^**^	−0.361^**^

** *p*<0.01 (two tailed).

The results from regressing age, gender, length of nursing work, personality, and grit on task performance are presented in Table 4. In the first step, age, gender, length of nursing work, and personality factors were used as independent variables and task performance as dependent variables for regression analysis. The results showed that length of nursing work (*β*=0.258, *p*=0.048), agreeableness (*β*=0.200, *p*<0.001), conscientiousness (*β*=0.241, *p*<0.001), and openness (*β*=0.134, *p*=0.001) were related to task performance of nurse. In the second step, when entering two factors of grit (consistency of interest and perseverance of effort) into the model, age (*β*=−0.294, *p*=0.021), length of nursing work (*β*=0.305, *p*=0.016), agreeableness (*β*=0.155, p<0.001), conscientiousness (*β*=0.140, *p*=0.002), openness (*β*=0.097, *p*=0.012), and perseverance of effort (*β*=0.261, *p*<0.001) were related to task performance of nursing. However, there was no statistical correlation between consistency of interest and task performance in the nurse sample.

**Table 4:** Hierarchical regression analysis of the impact of age, gender, length of nursing work, personality, and grit on task performance.

	Step 1			Step 2		
	*β*	*t*	*p*	*β*	*t*	*p*
Age	−0.238	−1.824	0.069	−0.294	−2.317	0.021^*^
Gender	0.059	1.727	0.085	0.055	1.655	0.098
Length of nursing work	0.258	1.984	0.048^*^	0.305	2.408	0.016^*^
Extraversion	−0.005	−0.121	0.903	−0.038	−0.960	0.338
Agreeableness	0.200	4.926	<0.001^***^	0.155	3.861	<0.001^***^
Conscientiousness	0.241	5.565	<0.001^***^	0.140	3.099	0.002^**^
Neuroticism	−0.001	−0.026	0.979	−0.005	−0.107	0.914
Openness	0.134	3.448	0.001^**^	0.097	2.533	0.012^*^
Consistency of interest				0.020	0.515	0.607
Perseverance of effort				0.261	6.367	<0.001^***^
*R* ^2^		0.209			0.255	
Δ*R*^2^					0.046	
*F*	23.133^***^			23.905^***^		

* *p*<0.05;

** *p*<0.01;

*** *p*<0.001 (two tailed).

### Measurement Invariance

Multi-group confirmatory factor analysis was conducted to test the MI across nurses working in different hospitals (general hospital and psychiatric hospital). The comparison of models tested in MGCFA was shown in Table 5. The higher-level test of MI could be executed only after the lower-level test passes (*p*<0.05). It passed four tests in turn in this study: configural invariance, metric invariance, scalar invariance, and strict invariance and all values of *p* were not less than 0.05. The results showed that Grit-S had good measurement invariance among nurses in general hospitals and psychiatric hospitals.

**Table 5:** The indices of model fit of Grit-S analyzed by MGCFA.

Model	*χ* ^2^	df	Δ*χ*^2^	Δdf	AIC	BIC	*p*
Configural	81.240	38			13,033	13,261	
Metric	84.893	44	3.6527	6	13,024	13,225	0.72
Scalar	97.674	50	12.7810	6	13,025	13,198	0.05
Strict	103.550	58	5.8758	8	13,015	13,152	0.66

MGCFA, multi-group confirmatory factor analysis; df, degree of freedom; AIC, Akaike information criterion; and BIC, Bayesian information criterion.

## Discussion

The Grit-S was developed from the original Grit Scale (Duckworth et al., 2007; 12-items version) and maintained the same two-factor structure. Compared with the original version, Grit-S has fewer items (eight-items version) and still maintains good reliability and validity, and it is widely used and translated into multilingual versions, such as Polish version (Wyszyńska et al., 2017), Spanish version (Arco-Tirado et al., 2018), and Czech version (Schmidt et al., 2021). Li et al. (2018b) translated it into Chinese and evaluated its psychometric properties among Chinese adolescents. Following previous studies, this study expanded the applicable population of Grit-S and verified the validity of construct and the acceptable reliability in the Chinese nurse sample.

The convergent validity and criterion-related validity also were proved, as shown in Tables 3 and 4. It is worth noting that this study found that perseverance of effort was related to task performance in sample of nurses. However, the relationship between consistency of interest and task performance has not been found. The work performance might be more affected by the perseverance of effort facet of grit than the consistency of interest facet. A meta-analysis based on 66,807 participants showed that perseverance of effort could explain the variance in academic performance even after controlling for some variables (Credé et al., 2017). Moreover, good measurement invariance across nurses in general hospitals and psychiatric hospitals was found.

Although the two-dimensional structure (consistency of interest and perseverance of effort) of grit was significant, item 2 (*setbacks do not discourage me*) was noteworthy because its low standardized factor loading (0.25) on perseverance of effort. In the study of Duckworth and Quinn in 2009, it was found that the standardized factor loading of this item was 0.37, which was not ideal. The same results have appeared in Polish version (standardized factor loading=0.33; Wyszyńska et al., 2017), Spanish version (standardized factor loading=0.27; Arco-Tirado et al., 2018), and Chinese version (standardized factor loading=0.34; Li et al., 2018b). This study also found that after removing item 2, the internal consistency of the factor (perseverance of effort) and the total Grit-S were improved, which increased from 0.682 and 0.751 to 0.772, respectively. Item 2 (*setbacks do not discourage me*) was not ideal in different cultural background. It might be caused by the expression difference. Specifically, the expression of “*setbacks do not discourage me*” might be inconsistent with daily expression in different cultural backgrounds, or it was not easy to be used by individuals in daily life. Whether item 2 (*setbacks do not discourage me*) belongs to the concept of grit or how to revise its expression needs further research.

More and more studies showed that grit played an important role in the cultivation of nurses (Halperin and Regev, 2021), the maintenance of nurses’ physical and mental health, the improvement of academic performance (Oducado, 2021), and the stability of nursing staff (Jeong et al., 2019). Adopting a widely used, simple and effective scale to measure nurses’ grit could not only improve the overall understanding of the concept, but also provide the possibility for the comparison of grit in different occupations. The professional dilemma faced by psychiatric nurses has always been worthy of attention. In addition, they will be exposed to more psychological training than nurses in general hospitals. This contradiction might interfere psychiatric nurses’ understanding of grit, which is also related to measurement accuracy of grit in nurse population. This study verified that Grit-S had excellent measurement invariance in psychiatric nurses and general hospital nurses and suggested that Grit-S could be an appropriate tool to measure the grit among Chinese nurses in the future.

The limitations of this study are as follows. First, the measurement bias comes from the self-report questionnaire, that is, the inaccurate report of participants. Second, the measurement invariance test was conducted only across nurses in psychiatric hospitals and general hospitals. In measuring invariance, more nursing subspecialties should be involved in the future. Finally, the Chinese version of Grit-S used in this study did not undergo the back-translation process.

## Conclusion

Grit is an essential trait for the challenging profession of nursing. This study demonstrated the Grit-S is a sound scale in measuring grit among Chinese nurses. In addition, through the measurement invariance test, it was suggested that different sub-professional nurses might have the consistent understanding of grit. This study is a fair foundation for the study on nurse grit in the future.

## Data Availability Statement

The raw data supporting the conclusions of this article will be made available by the authors, without undue reservation.

## Ethics Statement

The studies involving human participants were reviewed and approved by the Ethics Committee of Chengdu Fourth Hospital. The patients/participants provided their written informed consent to participate in this study.

## Author Contributions

CH, DW, and YY were involved in all aspects of the study from design, analysis, interpretation of data, and preparation of the manuscript. LuY and LeY were involved with the interpretation of data and preparation of the manuscript. All authors contributed to the article and approved the submitted version.

## Funding

This work was supported by the Sichuan Science and Technology Program (grant 2018JY0306) and the National Natural Science Foundation of China (grant 82001444).

## Conflict of Interest

The authors declare that the research was conducted in the absence of any commercial or financial relationships that could be construed as a potential conflict of interest.

## Publisher’s Note

All claims expressed in this article are solely those of the authors and do not necessarily represent those of their affiliated organizations, or those of the publisher, the editors and the reviewers. Any product that may be evaluated in this article, or claim that may be made by its manufacturer, is not guaranteed or endorsed by the publisher.
